# Early postoperative weight loss predicts maximal weight loss after sleeve gastrectomy and Roux-en-Y gastric bypass

**DOI:** 10.1007/s00464-014-3829-7

**Published:** 2014-09-20

**Authors:** Sean Manning, Andrea Pucci, Nicholas C. Carter, Mohamed Elkalaawy, Giorgia Querci, Silvia Magno, Anna Tamberi, Nicholas Finer, Alberic G. Fiennes, Majid Hashemi, Andrew D. Jenkinson, Marco Anselmino, Ferruccio Santini, Marco Adamo, Rachel L. Batterham

**Affiliations:** 1 Department of Medicine, Centre for Obesity Research, Rayne Institute, University College London, Rayne Building, 5 University Street, London, WC1E 6JJ UK; 2 UCLH Centre for Weight Loss, Metabolic and Endocrine Surgery, University College London Hospitals, Ground Floor West Wing, 250 Euston Road, London, NW1 2PG UK; 3 National Institute of Health Research University College London Hospitals Biomedical Research Centre, London, W1T 7DN UK; 4 Obesity Center, University Hospital of Pisa, Pisa, Italy; 5 Queen Alexandra Hospital, Southwick Hill Road, Portsmouth, PO6 3LY UK; 6 Clinical and Experimental Surgery Department, Medical Research Institute, University of Alexandria, Hadara, Alexandria, 21561 Egypt; 7 Surrey Weight Loss Centre, St Anthony’s Hospital, North Cheam, SM3 9DW UK; 8 Bariatric Surgery Unit, University Hospital of Pisa, Pisa, Italy

**Keywords:** Obesity, Bariatric surgery, Roux-en-Y gastric bypass, Sleeve gastrectomy, % weight loss, BMI

## Abstract

**Background:**

Previous studies show that ‘poor responders’ to Roux-en-Y gastric bypass (RYGBP) may be identified on the basis of early postoperative weight loss. Early identification of poor responders could allow earlier provision of postoperative behavioural and/or intensive lifestyle interventions and enhance their maximal weight loss. Our aim was to investigate whether early postoperative weight loss predicts the maximal weight loss response after RYGBP and sleeve gastrectomy (SG).

**Methods:**

We undertook a retrospective cross-sectional study of 1,456 adults who underwent either RYGBP (*n* = 918) or SG (*n* = 538) as a primary procedure in one of two European centres. Postoperative weight loss was expressed as weight loss velocity (WLV) and percentage weight loss. Linear regression analyses were performed to determine the association of early postoperative weight loss with maximal %WL, including adjustment for baseline variables.

**Results:**

There was marked variability in maximal %WL following both RYGBP (mean 32.9 %, range 4.1–60.9 %) and SG (mean 26.2 %, range 1.1–58.3 %). WLV 3–6 months postoperatively was more strongly associated with maximal %WL (*r* ^2^ = 0.32 for RYGBP and *r* ^2^ = 0.26 for SG, *P* < 0.001 for both) than either WLV 0–6 weeks or 6 weeks to 3 months postoperatively (*r* ^2^ = 0.14 and 0.10 for RYGBP, respectively; *r* ^2^ = 0.18 and 0.21 for SG, respectively; *P* < 0.001 for all). Multiple linear regression analysis, including baseline variables of age, sex, preoperative BMI, type 2 diabetes, ethnicity, and bariatric centre, revealed that 3–6 month WLV was an independent predictor of maximal %WL in both SG and RYGBP groups (standardised *β*-coefficients 0.51 and 0.52, respectively; *P* < 0.001 for both).

**Conclusions:**

There is a marked variability in weight loss response following RYGBP and SG. Early postoperative weight loss can be used to identify patients whose predicted weight loss trajectories are suboptimal. Early targeting of poor responders with more intensive postoperative lifestyle and behavioural support could potentially enhance their weight loss response.

Bariatric surgery is currently the most effective weight loss intervention for patients with severe obesity, is cost-effective, and significantly reduces morbidity and mortality [1, 2]. Roux-en-Y gastric bypass (RYGBP) is considered the ‘gold standard’ procedure with the most robust long-term clinical outcome data [1]; however, sleeve gastrectomy (SG) is fast attaining the status of a valid alternative to RYGBP [3], overtaking adjustable gastric banding (AGB) in the hierarchy of bariatric surgical interventions [4]. All three bariatric procedures lead to sustained weight loss and amelioration of obesity-related comorbidities that is unrivalled by medical interventions [5, 6]. There is, however, a wide variability in the weight loss response to RYGBP and AGB, though unknown for SG [7–9], with maximal weight loss occurring at 12–18 months postoperatively in the majority of patients [8–10].

Previous studies have identified preoperative clinical factors that are associated with reduced weight loss after bariatric surgery, although primarily for RYGBP. For example, higher baseline BMI, male sex, and older age are consistent predictors of suboptimal weight loss [7, 11–18]. Of note, relatively few studies have examined the effects of such factors in relation to SG [16]. However, these clinical factors explain only a small proportion of the observed variability in weight loss [7]. Given the lack of powerful preoperative predictors of weight outcomes, it is notable that ‘poor responders’ to RYGBP and AGB may be identified early in the postoperative period on the basis of early weight change patterns [19–21]. However, whether early postoperative weight loss also predicts long-term weight outcomes for SG is unknown. Importantly, postoperative behavioural or intensive lifestyle interventions improve weight loss after bariatric surgery [22–24], thus early identification of poor responders is an important focus for postoperative care. The aims of the study were thus to investigate whether early postoperative weight loss is an important predictor of the maximal weight loss response after RYGBP or SG, to identify early weight change patterns that are most predictive of maximal %WL, and to explore whether variability in weight loss after SG is similar to that observed after RYGBP.

## Methods

### Study subjects and design

This study was designed as a retrospective cohort study. Data were obtained by review of prospectively maintained electronic clinical data records and clinical case notes within two European university hospital bariatric surgery centres. Patients aged 18 or over, with a BMI ≥40.0 kg/m^2^, or ≥35.0 kg/m^2^ in the presence of at least one obesity-related comorbidity, who underwent either RYGBP or SG as a primary bariatric procedure at University College London Hospitals (UCLH) Centre for Weight loss, Metabolic and Endocrine Surgery, London, UK or at the Obesity Centre of the University Hospital of Pisa (UHP), Pisa, Italy were included in the study. In both centres, the decision for procedure selection was based on informed patient preference after standardised counselling including details, risks, and benefits of each procedure. Patients were advised to follow a liquid diet for 2 weeks postoperatively, followed by softer foods for 2 weeks, before resuming a solid diet thereafter. The postoperative follow-up schedule for patients in both centres comprised appointments at 6 weeks, then 3 monthly during the first year and 6 to 12 monthly thereafter, for a minimum of two postoperative years.

### Surgical technique: RYGBP

In both centres, RYGBP was performed using a laparoscopic, antecolic, antegastric RYGBP. At the UCLH centre, 30–40 ml gastric pouch was fashioned, and the alimentary limb was measured at 120 cm. The omentum was divided longitudinally, and a stapled jejunojejunal anastomosis was performed. In the UHP centre, a subcardial gastric pouch with a 30–50-ml capacity was created, and an enteroenterostomy was performed at 150 cm on the alimentary limb.

### Surgical technique: SG

In each centre, SG was performed using a standard laparoscopic technique. In the UCLH centre, the sleeve was created around the bougie using a laparoscopic stapler, 2.0 mm staple height on the gastric antrum and body and 1.8 mm staple height for the rest of the stomach, with staple line reinforcement. In the UHP centre, a four-fifths vertical gastrectomy was performed on a 34-French bougie using multiple reinforced 60-mm linear stapler firings starting 4–6 cm from the pylorus up to the gastroesophageal junction.

### Measures

Demographic, anthropometric, and clinical data were collected from electronic clinical data records. Age was defined as the difference between the date of birth and date of surgery. Baseline BMI was calculated from the weight and height measurements recorded on the day of surgery. Postoperative weight loss was determined relative to weight on the day of operation. Percentage weight loss (%WL) was chosen as the outcome measure for weight change, as %WL is less influenced by baseline BMI than % excess weight loss or BMI change, thereby facilitating a more sensitive identification of novel predictors of weight outcome [25]. Weight loss velocity (WLV) in the early postoperative period (0–6 months) was expressed as kg lost per week between postoperative follow-up appointments, i.e. during 0–6 weeks, 6 weeks to 3 months and 3–6 months.

### Statistical analysis

Baseline differences between patients in each bariatric centre were assessed using two-tailed *t* tests or *χ* ^2^ tests as appropriate. Linear regression analyses were performed to determine the association of early postoperative weight loss with maximal and 2-year %WL. Multiple regression analyses were performed with maximal %WL as the outcome measure, including adjustment for baseline variables. Using backward selection, variables with *P* < 0.05 were retained in the models. A receiver operating characteristic (ROC) curve was plotted in order to identify the optimal cutoff point for early postoperative WLV in predicting maximal %WL outcome. We chose a target %WL of 20 % for this analysis, as %WL not reaching this target is likely to represent a disappointment for the vast majority of patients [26]. The optimal cutoff value was determined using Youden’s index, i.e. by maximizing the point on the ROC curve furthest from the line of equality. Analyses were performed with Stata™ software, version 13 (StataCorp, TX, USA).

## Results

### Baseline patient characteristics

A total of 1,456 adults (Table 1) underwent either RYGBP (*n* = 918) or SG (*n* = 538) as a primary procedure. There were significant group differences in age, baseline BMI, and diabetes status between centres. In relation to the SG group, age was significantly higher in the UHP centre than the UCLH centre (*P* < 0.001) (Table 1). In relation to the RYGBP group, baseline BMI was significantly higher in the UHP centre than the UCLH centre (*P* < 0.001), whereas the proportion of patients with T2D (*P* = 0.03) was significantly higher in the UCLH centre than the UHP centre. There were no significant group differences in gender distribution between centres. Maximal weight loss data were calculated for 877 (95.5 %) patients who underwent RYGBP and 513 (95.3 %) patients who underwent SG, with weight data available for 715 (77.9 %) patients in the RYGBP group and 390 (72.5 %) patients in the SG group at the 2-year postoperative appointment.

**Table 1:** Baseline demographic, clinical, and anthropometric characteristics

	All		UCLH		UHP		*P*
	RYGBP	SG	RYGBP	SG	RYGBP	SG	
All	918	538	436	443	482	95	<0.001^a^
Women	711 (77 %)	393 (73 %)	350 (80 %)	319 (72 %)	361 (75 %)	74 (78 %)	0.75^a^
Men	207 (23 %)	145 (27 %)	86 (20 %)	124 (28 %)	121 (25 %)	21 (22 %)	
T2D	274 (30 %)	188 (35 %)	145 (33 %)	150 (34 %)	129 (27 %)	38 (40 %)	0.06^a^
Ethnicity							
White	824 (90 %)	439 (82 %)	342 (78 %)	344 (78 %)	482	95	
Other	94 (10 %)	99 (18 %)	94 (22 %)	99 (22 %)	–	–	
Mean age ± SD (years)	43.8 (10.6)	46.5 (11.1)	43.8 (11.9)	44.7 (10.4)	43.7 (10.1)	54.5 (10.5)	0.04^b^
Mean BMI ± SD (kg/m^2^)	48.3 (7.7)	49.8 (8.8)	46.0 (5.7)	50.0 (9.1)	50.3 (8.6)	48.6 (7.6)	<0.001^b^

^a^Procedure and sex distribution, and T2D status were compared between centres using Pearson’s *χ* ^2^ tests

^b^Comparisons of mean age and BMI between centres were performed using unpaired two-tailed *t* tests

### %WL following RYGBP and SG

**Fig. 1:**
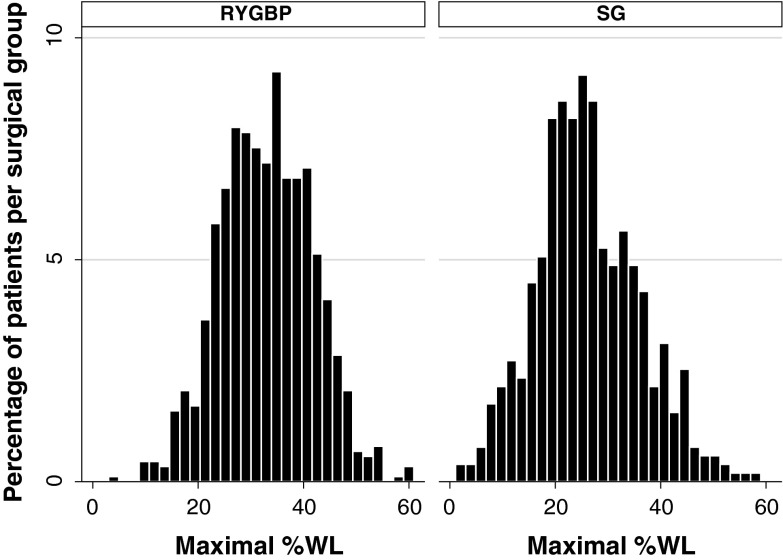
Histogram of maximal %WL for patients in RYGBP (*n* = 877) and SG (*n* = 513) groups

**Fig. 2:**
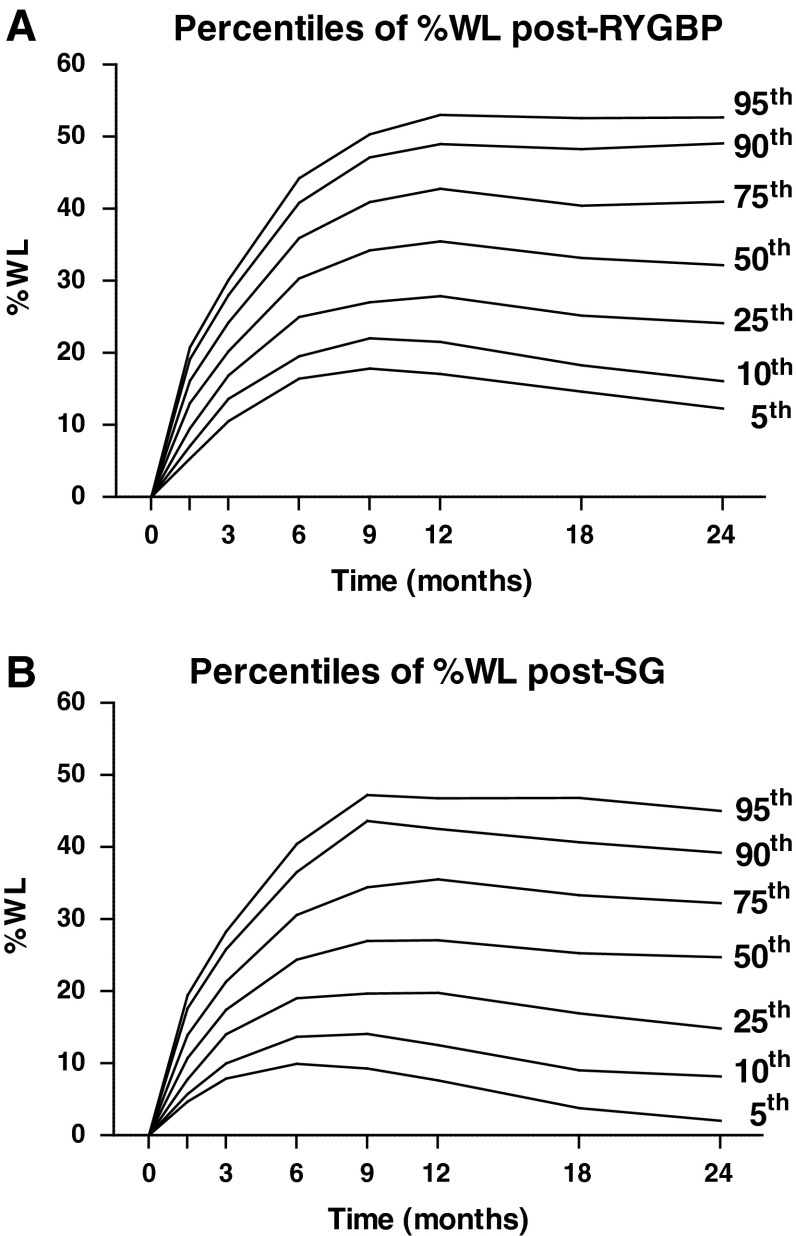
Normative charts of weight loss trajectories, based on percentiles of %WL at standard postoperative timepoints for patients in RYGBP (**A**) and SG (**B**) groups

There was a marked variability in maximal %WL (Fig. 1) following both RYGBP (mean 32.9 %, range 4.1–60.9 %) and SG (mean 26.2 %, range 1.1–58.3 %). Maximal %WL occurred at the 12-month follow-up appointment in approximately one-third of patients in both RYGBP and SG groups (Table 2). However, a higher proportion of patients in the RYGBP group experienced maximal %WL at the 24-month appointment, with lower proportions than the SG group at the 6- and 9-month appointments (*P* < 0.001). In order to visualize the distribution of weight loss trajectories, normative charts were constructed based on percentiles of %WL at postoperative timepoints and showed similar weight loss variability for both RYGBP and SG patients (Fig. 2).

**Table 2:** Timing of maximal %WL

Postoperative months	RYGBP		SG	
	*N*	%	*N*	%
6	78	**9**	70	**14**
9	96	**11**	88	**17**
12	324	37	188	37
18	175	20	100	19
24	204	**23**	67	**13**
*P* ^a^	<0.001			

^a^Distribution of patients with maximal %WL across postoperative timepoints was compared between procedures using a *χ* ^2^ test for trend—a higher proportion of RYGBP patients experienced maximal %WL at the 24-month appointment, with lower proportions than the SG group at the 6- and 9-month appointments (important differences in bold)

### Association of early postoperative %WL with maximal %WL

**Fig. 3:**
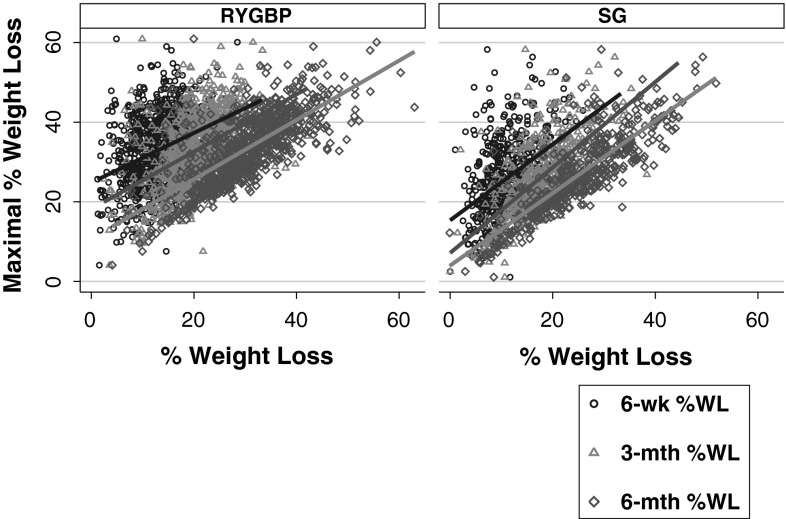
Scatterplots with maximal %WL as the outcome, and %WL at 6 weeks, 3 or 6 months as the predictor for patients in RYGBP and SG groups, with respective lines of best fit

In order to investigate whether early postoperative weight loss predicted the ultimate weight loss response, linear regression analyses were performed with maximal %WL as the outcome (Table 3). %WL at 6 weeks, 3 and 6 months was significantly associated with maximal %WL for patients in both RYGBP and SG groups (Fig. 3). The associations of early postoperative %WL with maximal %WL were stronger for SG than RYGBP at all three postoperative timepoints examined, but most notably at 6-week and 3-month assessments (Table 3).

**Table 3:** Strength of associations (*r* ^2^) between maximal %WL and early postoperative %WL, at 6 weeks, 3 and 6 months (left panel), or WLV during 0–6 weeks, 6 weeks to 3 months, and 3–6 months (right panel)

	*r* ^2^	*P*		*r* ^2^	*P*
SG			SG		
6-week %WL	0.21	<0.001	WLV 0–6 weeks	0.18	<0.001
3-month %WL	0.46	<0.001	WLV 6 weeks to 3 months	0.21	<0.001
6-month %WL	0.69	<0.001	WLV 3–6 months	0.26	<0.001
RYGBP			RYGBP		
6-week %WL	0.12	<0.001	WLV 0–6 weeks	0.14	<0.001
3-month %WL	0.23	<0.001	WLV 6 weeks to 3 months	0.10	<0.001
6-month %WL	0.53	<0.001	WLV 3–6 months	0.32	<0.001

### Association of early postoperative WLV with maximal and 2-year %WL

**Fig. 4:**
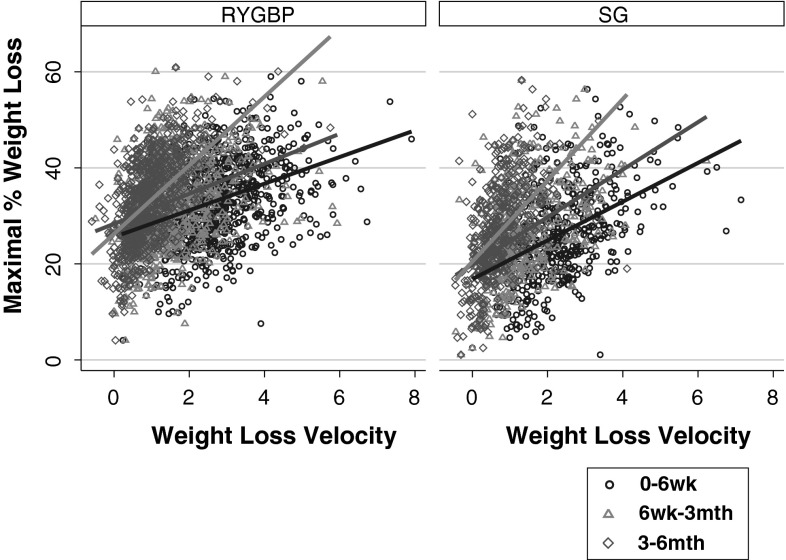
Scatterplots with maximal %WL as the outcome, and WLV during 0–6 weeks, 6 weeks to 3 months, or 3–6 months time intervals, as the predictor for patients in RYGBP and SG groups, with respective lines of best fit

Next, we examined the relative importance of weight loss during specific time intervals in the early postoperative period in relation to the maximal %WL achieved. In order to determine this, WLV during the 0–6 weeks, 6 weeks to 3 months, and 3–6 months postoperative time periods was used in linear regression analyses. We observed procedure-specific temporal differences in the strength of association between postoperative WLV and maximal %WL (Fig. 4). In the SG group, WLV during the 0–6 weeks and 6 weeks to 3 months postoperative periods predicted 18 and 21 % of the variability in maximal %WL respectively, increasing to 26 % for the 3–6 month period (Table 3). Whereas in the RYGBP group, WLV during the 0–6 week period and 6 weeks to 3 months period accounted for only 12 and 10 % of the variability in maximal %WL, respectively, increasing markedly to 32 % for the 3–6 month period (Table 3). Early postoperative WLV associations with 2-year %WL were comparable with those for maximal %WL (Table 4).

**Table 4:** Strength of associations (*r* ^2^) between 2-year %WL and WLV during 0–6 weeks, 6 weeks to 3 months, and 3–6 months

	*r* ^2^	*P*
SG		
WLV 0–6 weeks	0.08	<0.001
WLV 6 weeks to 3 months	0.29	<0.001
WLV 3–6 months	0.31	<0.001
RYGBP		
WLV 0–6 weeks	0.11	<0.001
WLV 6 weeks to 3 months	0.21	<0.001
WLV 3–6 months	0.40	<0.001

### Multiple linear regression analyses

Multiple linear regression analyses including baseline variables of age, sex, preoperative BMI, diabetes, ethnicity and bariatric surgery centre revealed that 3–6 months WLV, baseline BMI, and age were independent predictors of maximal %WL for both SG and RYGBP groups (Table 4). In addition, gender and T2D were independently associated with maximal %WL in the RYGBP group, while ethnicity and bariatric centre were independently associated with 1-year %WL in the SG group. However, in comparison with baseline variables, 3–6 month WLV was the strongest predictor of maximal %WL following RYGBP and SG (Table 5).

**Table 5:** Results of multiple regression analyses, after backward selection, with maximal %WL as the outcome measure, and expressed with standardised effect sizes (*β*-coefficient)

SG	Standardised *β*-coefficient	*P*	RYGBP	Standardised *β*-coefficient	*P*
WLV 3–6 months	0.51	<0.001	WLV 3–6 months	0.52	<0.001
Baseline BMI	−0.19	<0.001	Age	−0.09	0.003
Age	−0.18	<0.001	T2D	−0.08	0.005
Centre	0.16	<0.001	Baseline BMI	0.08	0.008
Ethnicity	0.09	0.015	Male sex	−0.07	0.008

**Fig. 5:**
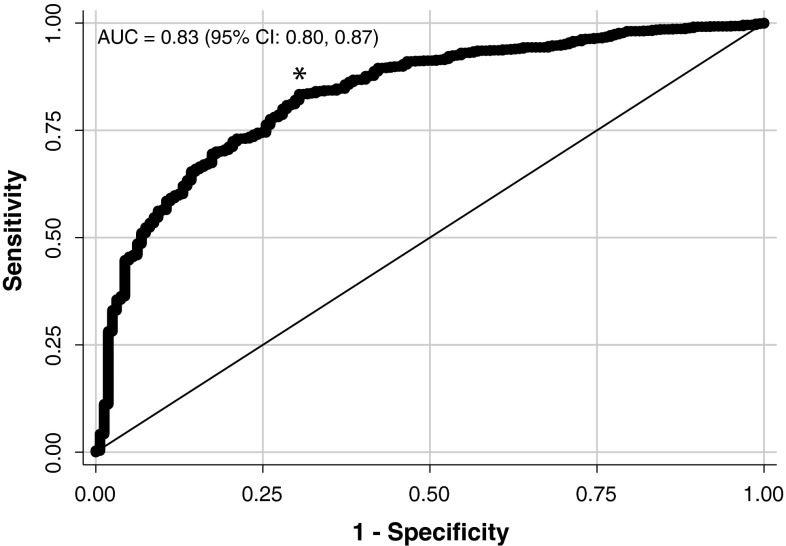
ROC demonstrating the ability of WLV during the 3–6 month time interval to predict maximal %WL ≥20 % expressed as area under curve (*AUC*). The inflection point (*asterisk*) corresponded to a sensitivity of 80 % and a specificity of 72 %

A ROC curve (Fig. 5) was constructed to determine the point at which 3–6 month WLV predicted maximal %WL (using a target %WL of 20 %) with the best sensitivity/specificity combination. The inflection point corresponded to a sensitivity of 80 % and a specificity of 72 %. Using this cutoff, which occurred at a WLV of 0.4667 kg/week (1 lb/week), the maximal %WL outcome was classified correctly for 79 % of patients.

## Discussion

The findings in our study provide novel insights into the weight loss response experienced both after RYGBP and SG. Patients who underwent either SG or RYGBP experienced a wide variability in weight loss during the first two postoperative years. In a particularly novel aspect of the study, the variability in maximal weight loss was shown to be strikingly similar between procedures. Furthermore, the results demonstrate that early postoperative weight loss is a key predictor of ultimate weight loss response, with a greater effect on outcome than several well-established baseline clinical factors such as preoperative BMI, age, sex, and diabetes.

Interestingly, our results show temporal procedure-specific differences in the relationship between early postoperative weight loss, expressed as %WL or WLV, and maximal weight loss. In the immediate postoperative period (6 weeks post-surgery), %WL is a stronger predictor of maximal weight loss in the SG group compared to the RYGBP group. Similarly, the strength of the association between %WL in the first three postoperative months and maximal weight loss in the SG group was approximately twice that observed in the RYGBP group. In order to identify which postoperative time period may best predict ultimate weight loss outcome, we examined WLV during specific postoperative time intervals, a concept which has been previously applied in a study of RYGBP outcomes [21]. WLV experienced in the 3–6 month postoperative period was a stronger predictor of maximal weight loss, compared to the earlier postoperative time intervals, for both SG and RYGBP groups. However, in the RYGBP group, the strength of the association with maximal weight loss was approximately threefold higher for the 3–6 month postoperative period compared to the earlier postoperative time intervals, but only approximately 25–50 % higher in the SG group. Taken together, these findings support the concept that both distinct and overlapping biological mechanisms underlie the benefits of SG and RYGBP [27, 28].

Our findings have several potential clinical implications for patients undergoing either SG or RYGBP. Firstly, focus on early postoperative WLV is an effective means of identifying patients whose weight loss is ultimately suboptimal. Our ROC analysis demonstrates that approximately four out of every five patients who lose less than a 1 lb a week during the 3–6 month postoperative period will not achieve a maximal %WL of more than 20 %. Such patients could be targeted for early postoperative behavioural or intensive lifestyle interventions known to improve weight outcome after surgery [22–24], thereby providing an opportunity to enhance their maximal weight loss. Our results provide a basis for randomized trials of behavioural or exercise interventions initiated early in the postoperative course for both RYGBP and SG patients.

Secondly, the wide variability in weight loss response, previously demonstrated only in RYGBP patients [8, 9], is similar for both procedures. Therefore, bariatric health care professionals should alert patients who are considering surgery to this inherent variability as part of the informed consent process. In this regard, our normative charts provide a useful reference for expected weight loss trajectories post-SG or RYGBP and are consistent with the results of a previous single-centre study in RYGBP patients [21]. The practice of providing advice to patients regarding expected weight loss based on an average narrow range [29] is likely to be counterproductive, ultimately leading unnecessarily to a sense of disappointment or failure for many patients [26]. Indeed, a poor weight loss response is likely to merely reflect an outcome at the lower tail of a normal distribution driven by a multitude of complex biological factors [7, 30]. Patients should be advised as such preoperatively and be relieved of any sense of blame if necessary in the postoperative setting.

Thirdly, our findings are consistent with the strong biological basis that underlies the benefits of bariatric surgery [31]. Indeed, the weight loss response to RYGBP is known to be highly heritable [32], suggesting that patients’ responses to bariatric surgery may, to a large extent, be predetermined by their genotype. Genome-wide association studies have demonstrated associations of common genetic variants with 1-year weight loss response in patients after RYGBP [30, 33]. However, personalized medicine, like in many clinical specialties, has thus far had limited clinical impact, if any, in the field of bariatric surgery [34]. In this light, our findings suggest that an individual’s maximal weight loss response to SG or RYGBP may be most practically predicted by tracking their actual weight change in the early postoperative period.

A potential limitation of our study is the focus on maximal weight loss. However, this is clearly an important outcome for patients who undergo bariatric surgery [26]. Moreover, weight regain subsequent to the maximal weight loss achieved is likely to reflect a completely different biological process from that governing the initial weight loss. Such weight regain is subject to a multitude of biological, psychological, and environmental influences, and remains poorly defined [35]. In order to address a definable research question, we focused on maximal weight loss response and not subsequent weight change. Prediction of late weight regain after bariatric surgery may be equally important; however, maximization of the initial weight loss response is clearly central to optimizing long-term outcome [8, 36]. Interestingly, we found that in addition to being associated with maximal %WL, early postoperative WLV was also associated with 2-year %WL. These findings suggest that early WLV predicts longer-term postoperative weight loss outcomes but longer-term studies are required to confirm this. Another interesting question not addressed by our study is whether early postoperative weight loss also predicts resolution of comorbidities. However, the benefits of bariatric surgery in ameliorating obesity-related comorbidities are in proportion to the weight loss achieved [36]. Finally, a further potential limitation is the difference in baseline patient characteristics between bariatric centres. In particular, the population of patients who underwent SG in the UHP centre was significantly different from SG patients in the UCLH centre (smaller sample size and older age). This difference may, in turn, have contributed to the finding that centre predicted maximal weight loss in the SG group but not the RYGBP group. This limitation was unlikely to affect the robust association of early postoperative weight loss with maximal weight loss, and we believe the study benefits from its multicentre dimension.

## Conclusions

Our results demonstrate that there is a wide variability in weight loss response after both RYGBP and SG. Moreover, patients who ultimately experience suboptimal weight loss after either procedure can be identified based on early postoperative weight loss, within the first six postoperative months and primarily during the 3–6 month postoperative period, offering the opportunity for adjunctive interventions that could enhance their weight loss response. Detailed characterization of weight loss trajectories also provides an impetus to enhance bariatric care pathways with a greater emphasis on promoting understanding of expected outcomes among clinicians and patients alike, as well as patient-centred approaches to maximizing the benefits of these highly effective interventions.
